# Is this real? Susceptibility to deepfakes in machines and humans

**DOI:** 10.1186/s41235-025-00700-y

**Published:** 2026-01-07

**Authors:** Didem Pehlivanoglu, Mengdi Zhu, Jialong Zhen, Aude A. Gagnon-Roberge, Rebecca K. Kern, Damon Woodard, Brian S. Cahill, Natalie C. Ebner

**Affiliations:** 1 Department of Psychology, University of Florida, 945 Center Dr, Gainesville, FL 32603 USA; 2 Florida Institute for National Security, University of Florida, 601 Gale Lemerand Dr, Gainesville, FL 32611 USA; 3 Department of Electrical and Computer Engineering, University of Florida, 968 Center Dr, Gainesville, FL 32603 USA; 4 Center for Cognitive Aging and Memory, McKnight Brain Institute, University of Florida, 1149 Newell Dr, Gainesville, FL 32610 USA

**Keywords:** Deepfakes, Deception, Artificial intelligence, Machine learning, Confidence, Analytical thinking, Truth bias

## Abstract

Deepfakes are synthetic media created by deep-generative methods to fake a person’s audio-visual representation. Growing sophistication of deepfake technology poses significant challenges for both machine learning (ML) algorithms and humans. Here we used real and deepfake static face images (Study 1) and dynamic videos (Study 2) to *(i)* investigate sources of misclassification errors in machines, *(ii)* identify psychological mechanisms underlying detection performance in humans, and *(iii)* compare humans and machines in their classification decision accuracy and confidence. Study 1 found that machines achieved excellent performance in classifying real and deepfake images, with good accuracy in feature classification. Humans, in contrast, experienced challenges in distinguishing between real and deepfake static images. Their classification accuracy was at chance level, and this underperformance relative to machines was accompanied by a truth bias and low confidence for the detection of deepfake images. Using dynamic video stimuli, Study 2 found that performance of machines was near chance level, with poor feature classification. Further, machines showed greater lie bias and reduced decision confidence relative to humans who outperformed machines in the detection of video deepfakes. Finally, Study 2 revealed that higher analytical thinking, lower positive affect, and greater internet skills were associated with better video deepfake detection in humans. Combined, the findings across these two studies advance understanding of factors contributing to deepfake detection in both machines and humans; and can inform intervention toward tackling the growing threat from deepfakes by identifying areas of particular benefit from human-AI collaboration to optimize the detection of deepfakes.

**Supplementary Information:**

The online version contains supplementary material available at 10.1186/s41235-025-00700-y.

## Introduction

Significant advances in artificial intelligence (AI) have resulted in the use of sophisticated technology for producing manipulated media and has led to the emergence of *deepfakes* (for a review, see Nightingale & Wade, 2022). Deepfakes are algorithmic manipulations which are typically synthesized using generative adversarial networks, a type of machine learning that works by pitting two neural networks (a generator and a discriminator) against one another in an iterative back-and-forth process, to create any type of fake image, video, or audio (Tong et al., 2020). While deepfake technology presents numerous creative and entertainment possibilities for education, arts, and science, it also raises significant ethical, legal, and societal concerns, ranging from advertising to national security. In particular, deepfakes constitute a novel deception tactic to fake someone’s entire audio-visual representation for spreading false information (Seow et al., 2022; Ternovski et al., 2022; Zhang, 2022) and are effectively harnessed in social engineering (Vaccari & Chadwick, 2020; Westerlund, 2019).

The growing presence of deepfakes to manipulate public opinion on social and news platforms (Fallis, 2021; Vaccari & Chadwick, 2020) has led to computer science and psychology research into investigating machine and human performance for detecting deepfakes (Groh et al., 2022; Karras et al., 2020; Montserrat et al., 2020; Nightingale & Farid, 2022). Importantly, these lines of research have been almost exclusively focused on deepfake detection performance, with factors that contribute to the ability to detect deepfakes in machines and humans still poorly understood. Further, this past research has mostly been conducted in isolation in each discipline and a direct comparison between machine and human performance has not been conducted yet. To fill these research gaps, this project identified sources of misclassification errors in machines *(Aim 1)*, determined psychological mechanisms of deepfake detection in humans *(Aim 2)*, and directly contrasted machine and human performance in discernment of real and deepfake visual stimuli *(Aim 3)* across two studies, one employing static face images (Study 1) and one employing dynamic videos (Study 2). Next, we review the work leading to these central research aims.

### Machine Detection of Deepfakes

Deepfake images are typically synthesized using generative models that apply a replication process for the generation of new samples based on training data. Upon effective training, these models allow image synthesis, style transfer, and face-swapping. Among the multitude of existing generative models, generative adversarial networks (GANs; Goodfellow et al., 2014) are highly regarded for their ability to produce high-quality, high-resolution images. Detecting image deepfakes is a rapidly evolving field within computer vision and digital forensics. Generative models, however, often leave distinctive "fingerprints’’ on deepfakes, leading to the development of machine learning (ML) algorithms designed to identify and categorize such model-specific artifacts (Durall et al., 2019; Yu et al., 2021). In particular, Convolutional Neural Networks (CNNs) are trained on extensive datasets of real and fake images to learn distinguishing features. CNNs have demonstrated considerable promise in detecting deepfakes, with notable examples including ShallowNet (Tariq et al., 2018), ResNet-50 (Wang et al., 2020), Inception (Suratkar et al., 2020), Xception (Rössler et al., 2019; Suratkar et al., 2020), and MobileNet (Suratkar et al., 2020). CNN-based ML algorithms for image deepfake detection have reported accuracy rates between 83 and 100% (Afchar et al., 2018; Tolosana et al., 2020).

Video deepfakes often involve swapping of faces from source to target videos. Variational Autoencoders (VAEs; Kingma & Welling, 2014) are one of the methods frequently employed for face-swapping due to their proficiency in learning disentanglement within the data (Korshunova et al., 2017; Natsume et al., 2018). As for detecting video deepfakes, there are two primary methods. The first method involves CNNs, which follow a similar process as for image deepfake detection: each frame of an individual video from a training set of videos is processed by CNNs for final classification. CNNs have achieved video deepfake detection accuracies between 80 and 90% (Afchar et al., 2018; Sambhu & Canavan, 2020). Notably, the Xception network (Rössler et al., 2019) excels in learning complex data representations (i.e., face detection) and offers advantages such as efficiency and reduced susceptibility to overfitting. The second method leverages biometric and biological features for detection, as current deepfake technologies still struggle to accurately replicate such features. In particular, this approach includes analyzing facial features (Matern et al., 2019), eye blink patterns (Jung et al., 2020; Li et al., 2018), eye movements (Gupta et al., 2020), head poses (Yang et al., 2018), consistency of facial geometry (Tursman et al., 2020), facial expressions (Agarwal et al., 2020), lip syncing (Korshunov & Marcel, 2021), and biological signals from facial regions (e.g., photoplethysmography, head motion-based ballistocardiogram; Ciftci et al., 2020). Performance in distinguishing real videos from deepfakes using these feature-based ML algorithms ranges from 50 to 96%, but requires high-quality, high-resolution data for biometric feature extraction.

Growing evidence suggests variation in performance of different ML algorithms in detecting deepfake images and videos, but what contributes to this variation is not yet well understood. Going beyond existing work, here we determine factors that underlie machines’ ability to spot real and deepfake material (Aim 1). In particular, currently limited is understanding of how and why particular pieces of content get misclassified. Misclassifications are typically caused by biases within detection systems, such as training data bias, algorithmic bias, cultural and contextual bias, and/or performance discrepancies. We adopted a fine-grained approach by employing feature space analysis (Kulis, 2013) which allowed us to examine and compare two different ML algorithms regarding their classification accuracy of static face images (Study 1) and dynamic videos (Study 2). Analyzing feature detection performance will allow identification of misclassification sources by different ML algorithms, which is crucial for improving the feature selection capacity of these algorithms.

### Human Deepfake Detection

According to a recent national survey, a significant portion of Americans (63%) reported that made-up or altered images or videos create ‘‘a great deal of confusion’’ about the basic facts of current issues and events (Gottfried, 2019). Some studies employing static face images to determine discrimination ability between deepfake and real faces found that human performance was not better than chance (Miller et al., 2023; Nightingale & Farid, 2022; Rossi et al., 2023; Shen et al., 2021), with deepfake faces typically perceived as more real (Miller et al., 2023; Shen et al., 2021; Tucciarelli et al., 2022) and trustworthy (Nightingale & Farid, 2022) than real faces. Other studies demonstrated that while above chance, mean deepfake face detection accuracy in humans ranged from 60 to 65% only (Bray et al., 2023; Hulzebosch et al., 2020); and this performance was accompanied by participants’ overconfidence in their ability to detect deepfake faces (Bray et al., 2023; Miller et al., 2023). In brief, these findings reveal that humans are frequently fooled by deepfake faces and cannot reliably distinguish them from real faces.

There are also studies on human detection for dynamic video deepfakes, which vary widely regarding detection accuracy (from 58 to 89%; (Groh et al., 2022; Josephs et al., 2024; Köbis et al., 2021; Nas & de Kleijn, 2024; Somoray & Miller, 2023). For example, using deepfake videos pre-categorized based on subjective ratings of difficulty, one study found that participants detected “easy” video deepfakes with 71% accuracy whereas performance dropped to 25% for “very difficult” videos, indicating below chance-level performance for humans for high-quality deepfakes (Korshunov & Marcel, 2021). Another study found that overall detection accuracy for deepfake videos in humans was above chance (58%; Köbis et al., 2021). Thus, taken together, while human detection for static image deepfakes appears to be at chance, detection of at least some video deepfakes can be relatively good.

Currently less understood are individual differences in the ability to detect deepfakes. The limited research on this topic suggests that individuals with prior experience with histological images (i.e., microscopic images of tissues) were better able to distinguish between artificially generated and genuine histological samples than individuals without prior experience (Hartung et al., 2024). Also, somewhat counterintuitive, belief in conspiracy theories was positively correlated with deepfake video detection (Nas & de Kleijn, 2024). While informative, these studies have, however, failed to consider a larger spectrum of psychological factors that may contribute to deepfake detection ability. To fill this research gap, the current paper investigated interindividual differences in cognitive and socioemotional processing as well as experience and comfortability with the internet in their influence on deepfake detection accuracy in humans (Aim 2) for static face images (Study 1) and dynamic videos (Study 2). Investigation of these factors will inform the psychological mechanisms in deepfake detection, which can guide the development of interventions to reduce deception via deepfakes.

In particular, we assessed the following psychological variables:

**Cognitive Processing.** According to Dual-Process Theory (De Neys, 2012; Kahneman, 2011; Stanovich, 2009), individuals engage in two main routes of information processing: a quick, intuition-based route and a slow, deliberate route. While the intuition-based route leads to faster decision making, it is associated with low analytical reasoning and relies on cognitive heuristics. The slower route, in contrast, is associated with high analytical thinking and allows deliberation of information, often leading to less error-prone decision making. Indeed, research has consistently shown that individuals higher in analytical thinking were better at detecting misleading information (e.g., fake news; Pehlivanoglu et al., 2021, 2022; Pennycook & Rand, 2021). Further, need for cognition, which refers to the tendency to enjoy and engage in effortful and systematic thinking (Cacioppo et al., 1984), has been positively correlated with information seeking (Juric, 2017) and decision-making competence (Ding et al., 2020). Individuals with higher need for cognition are more willing to invest cognitive effort to solve demanding tasks and employ an elaborated information processing style instead of a heuristic processing style (Cacioppo et al., 1996; Verplanken et al., 1992). Also, individuals with higher need for cognition demonstrated greater skepticism toward information shared on social media (Tsfati & Cappella, 2003; Vraga & Tully, 2021). Based on this literature, we measured *analytical thinking* and need *for cognition* in their contributions to deepfake detection.

**Socioemotional Processing.** Affect has been shown to impact deception detection, though the direction of this effect is somewhat unclear (Ebner et al., 2020; Forgas & East, 2008; see also Ebner et al., 2023 for a summary). For example, individuals with greater feelings of sadness and distress (dysphoric mood) compared to non-dysphoric individuals were better at lie detection (Lane & DePaulo, 1999). Similarly, negative affect increased, while positive affect decreased skepticism, deception detection, and ambiguity (Matovic et al., 2014; but see LaTour & LaTour, 2009). Further, heightened emotionality (in the form of both increased positive and negative affect) was associated with worse fake news detection (Martel et al., 2020). Additionally, interoceptive awareness, which reflects the ability to read one’s inner bodily state (Bogaerts et al., 2022; Mehling et al., 2009), has been associated with better deception detection (Gunderson & Brinke, 2022; Heemskerk et al., 2024; ten Brinke et al., 2019). Based on these findings, we measured *affect* and *interoceptive awareness* in their contributions to deepfake detection.

**Experience and Comfortability with the Internet.**Having relevant skills and experience with the internet and online materials may influence the ability to detect deception. For instance, time spent on social media was negatively correlated with believing fake news (Halpern et al., 2019) and positively with the detection of deepfake videos (Nas & de Kleijn, 2024). Somewhat counterintuitive, one study found that self-reported IT affinity was not related to deepfake detection in both individuals with an IT background and non-professionals (Sütterlin et al., 2022). These previous studies, however, have not considered a broader set of internet and technology related skills that may contribute to the detection of deepfakes. Thus, going beyond existing literature, we measured self-reported *digital literacy* (i.e., internet skills) and *power usage* (i.e., mastery of technology use) in their contributions to deepfake detection.

### Human versus Machine Performance in Deepfake Detection

Currently, research from computer science on deepfake detection in machines is not well integrated with research on deepfake detection in humans. One exception is Groh et al. (2022) who directly compared human and machine performance and found comparable accuracy. Their study, however, only examined deepfake videos (not static images) and some videos involved familiar actors (i.e., political figures), which may have affected detection performance. Here we employed both static face images (Study 1) and dynamic videos (Study 2) of unfamiliar individuals and directly compared performance of the leading ML algorithm (i.e., the better performing ML algorithm among the two compared under Aim 1) with human performance **(Aim 3).** Findings from this work can generate insight into whether machines outperform humans in classification accuracy and confidence and increase knowledge about the nature of decision biases in machines and humans, to inform development of optimized human-AI collaboration in deepfake detection.

## Study 1

### Participants

Study 1 recruited 2418 undergraduates through the Department of Psychology’s SONA system. Of those, 183 who did not continue the study past consenting and 32 who had missing data on one or more of the variables of interest were removed from analysis. The final analysis sample comprised 2203 participants (Age range: 18–58 years, *M* = 19.64, *SD* = 3.18; 75% female).

### Measures

**Image Rating Task.** Participants were asked to rate the veracity of each of 200 faces on a scale from 1 (*Fake*) to 10 (*Real*) to balance simplicity with sufficient range while minimizing fatigue. Each face image was displayed alongside the response scale. To ensure that participants took time to view the stimuli rather than quickly advancing with a keypress, responses were disabled for the first 3 secs. After this initial viewing period, the face image remained on the screen with the response scale, and participants were then able to enter their response using the keyboard.

Real images were 300 human face images randomly selected from the Flickr-Faces-HQ (FFHQ) dataset (Karras et al., 2019), which contains 70,000 high-quality images (1024 × 1024 resolution) that vary in age, gender, ethnicity, and image background. The final set of real face images were crawled from Flickr, then aligned and cropped to ensure they contained only one face. To generate deepfake face images, we used a pre-trained StyleGAN2 network released by NVIDIA (Karras et al., 2020), which was initially trained on the FFHQ dataset. With the pre-trained network, we generated a large set of deepfake images from random noise signals and finalized it into a set of 300 deepfake images by removing images containing artifacts (e.g., warping artifacts). The StyleGAN2 algorithm enables intuitive, scale-specific control of the synthesizing process via an automatically learned, unsupervised separation of high-level attributions (e.g., pose and identity when trained on human faces) and stochastic variation in the synthesized images (e.g., freckles, hair, accessories). For equal numbers of real vs. deepfake images by gender, deepfake images were first classified as male vs. female using a deep-learning based classification algorithm, then cross-validated via manual selection to exclude images with interference and/or warping artifacts.

We compiled three sets of 200 stimuli each by randomly selecting 100 real and 100 deepfake images from the larger pool we had created, with face gender balanced within each set and image type (real vs. deepfake). Final image sets are achieved in the OSF repository (https://osf.io/qhm3y/?view_only=bdc41a53bf7a4367bde6951372d9c932). A third of participants were assigned to view one of the three sets, respectively, for counterbalancing, with face presentation order within each set randomized.^1^

**Cognitive Reflection Test (CRT).** Analytical thinking was assessed via the CRT (Frederick, 2005), which contains both numerical and logical propositions that have an intuitive and an analytical answer. For example, *“A bat and a ball cost $1.10 in total. The bat costs $1.00 more than the ball. How much does the ball cost? _____ cents.”* Individuals who rely on intuition respond with the intuitive answer (10 cents), whereas individuals who rely on effortful thinking respond with the analytical answer (5 cents).

Validity of the CRT is affected by familiarity with the items (Haigh, 2016) as well as number of scale items (Toplak et al., 2014). Here, we used a 7-item version, which consisted of three items from Shenhav et al. (2012) and four items from Thomson and Oppenheimer (2016). An example item was: *“The ages of Mark and Adam add up to 28 years total. Mark is 20 years older than Adam. How many years old is Adam?”.* Participants with high analytical thinking overcome the impulse to give the intuitive (incorrect) answer of *8 years old* and instead give the analytical (correct) answer of *4 years old.* We calculated sum scores across the 7 items, with higher CRT scores reflecting greater analytical thinking.

**Need for Cognition (NFC).** The NFC scale is a self-report questionnaire (Cacioppo & Petty, 1982) assessing how much an individual engages in and enjoys thinking or cognitively demanding tasks. We used a short version of the scale containing 18 items (Cacioppo et al., 1984). Each item consists of a statement, e.g. “*I would prefer complex to simpler problems*”, and participants score themselves on a scale from 1 (*Extremely uncharacteristic*) to 5 (*Extremely characteristic*). We calculated the mean across all 18 items, with higher NFC scores reflecting greater need for cognition.

**Positive and Negative Affect (PANAS)** We administered the 20-item PANAS (Watson et al., 1988), an affect assessment that contains 20 adjectives. We also included six additional adjectives to capture hedonic balance (Röcke et al., 2009). For each item, participants were asked “*To what extent do you feel [emotion adjective] right now?*” and used a scale from 1 (*Very slightly or not at all*) to 5 (*Extremely*) to evaluate each adjective (e.g., *excited*, *happy*, *afraid*, *alert*; 13 positive and 13 negative adjectives). We calculated the mean across positive adjectives and negative adjectives, with higher scores reflecting more positive affect and more negative affect, respectively.

**Multidimensional Assessment of Interoceptive Awareness Version 2 (MAIA-2).** MAIA-2 (Mehling et al., 2018) is a 37-item self-report questionnaire that measures awareness of bodily sensations. The scale is composed of 8 subscales measuring different aspects of interoception (i.e., noticing, not-distracting, not-worrying, attention regulation, emotional awareness, self-regulation, and body listening). Each subscale has Likert-type items, with response options ranging from 0 (*Never*) to 5 (*Always*). Sample items are, “*When I am tense, I notice where the tension is located in my body.”,* “*I can notice an unpleasant body sensation without worrying about it.”,* and “*I notice that my body feels different after a peaceful experience.*” We calculated the mean across all 37 items, with higher MAIA-2 scores reflecting greater interoceptive awareness.

**Digital Literacy Scale (DLS).** The DLS is a 21-item inventory that evaluates an individual’s familiarity with computer and internet elements (Hargittai, 2009). The current study used a modified version with updated internet terms (Guess & Munger, 2023). For each item, participants reported their level of understanding of various computer and internet elements (e.g., *phishing, tagging, selfie)* on a scale ranging from 1 (*No understanding*) to 5 (*Full understanding*). We calculated the mean across all 21 items, with higher scores reflecting greater understanding of digital media.

**Power User Scale (PUS).** The PUS (Sundar & Marathe, 2010) is a 12-item inventory that assesses mastery of information technology based on prior experience, expertise, and self-efficacy. The scale consists of two sub-scales, each with 6 items that were evaluated on a scale from -4 (*Strongly disagree*) to + 4 (*Strongly agree*). One subscale captures low (e.g., “*I think most technological gadgets are complicated to use*”) vs. high (e.g., “*I often find myself using many technological devices simultaneously”*) frequency of technology use. The other subscale captures low (e.g., “*I prefer to ask friends how to use any new technological gadget instead of trying to figure it out myself*”) vs. high (e.g., “*I would feel lost without information technology*”) comfortability with technology use. We calculated the mean across both subscales (all 12 items), with higher PUS scores reflecting greater power usage (i.e., expertise, experience, and efficacy in technology use).

### Procedure

All procedures and measures were approved by the University of Florida Institutional Review Board (IRB# 202,102,022). Participants completed this study remotely through Qualtrics (https://www.qualtrics.com/). Prior to study enrollment, all participants consented electronically to participate. Participants then completed the Image Rating Task, CRT, NFC, MAIA-2, PANAS, DLS, PUS, and a brief demographic questionnaire, in this order. The study took approximately 100 min and participants were reimbursed with SONA credits upon completion.

### Analyses and Results

All de-identified datasets and analysis scripts used in Study 1 are available on the OSF repository (https://osf.io/qhm3y/?view_only=bdc41a53bf7a4367bde6951372d9c932).

#### Machine Performance

To measure how well a machine could detect face image deepfakes, we chose two different ML algorithms, previously shown to be efficient in identifying specific artifacts existing in GAN-generated deepfake images (Mirsky & Lee, 2021; Verdoliva, 2020). The first approach applied a CNN (using a pre-trained ResNet-50 network; Wang et al., 2020); the second involved Frequency Domain Analysis (FDA; using a pre-trained Support Vector Machine; Durall et al., 2019) for extraction of frequency characteristics from images to distinguish real and deepfake images.^2^ These ML algorithms generated predicted labels as outcome variable. Predicted labels were either 0 = Deepfake face or 1 = Real face and reflected the classification of each face type. The CNN approach yielded 97% accuracy in distinguishing real and deepfake images, whereas the FDA approach resulted in 79% accuracy.

**Fig. 1:**
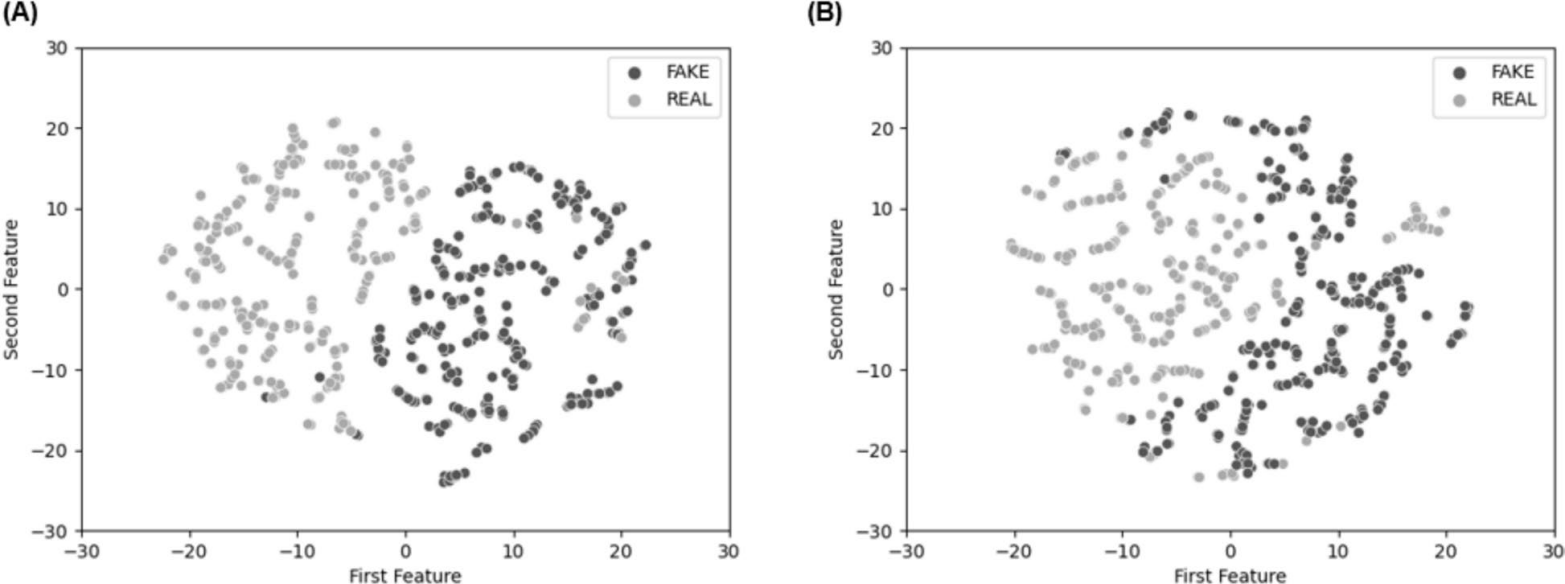
2-D visualization of latent features from **A** Convolutional Neural Network (CNN) and **B** Frequency Domain Analysis (FDA). Real images are shown in gray, deepfake images in black

To understand the source of misclassification underlying image detection performance of these two ML algorithms, we used feature visualization. This approach compared feature detection of the two ML algorithms in their classification accuracy of static face images. Specifically, this technique involved reducing the dimensionality of features for a 2D visualization of features using t-distributed stochastic neighbor embedding (t-SNE; van der Maaten et al., 2008). After applying feature analysis for both ML approaches, the decision boundary of the CNN approach (Fig. 1A) was more clearly defined than that of the FDA approach (Fig. 1B), resulting in higher classification accuracy of features in image deepfakes in the CNN than the FDA approach. One reason for the difference in performance could be that the CNN approach used persistent and distinctive features from both real and fake images, while the FDA approach only leverages features within the deepfake images, causing features in real images to get misclassified.

#### Human Performance

For analysis of the human data, we first assessed the face rating data for normality (Shapiro–Wilk tests; Shapiro & Wilk, 1965) and variance homogeneity (F-test; Snedecor & Cochran, 1989). The Shapiro–Wilk tests indicated significant deviations from normality for both real and fake faces (*W*s > 0.89, *p*s < .001). Additionally, an F-test revealed unequal variances between real and fake faces (*F*(175,099, 175,099) = 0.90, *p* < .001). Given these violations, we employed non-parametric AUC (Area Under the Receiver Operating Characteristic Curve; Hanley & McNeil, 1983) scores derived from participants’ continuous ratings as an index of sensitivity in discriminating between real and deepfake face images. AUC scores provide a robust and assumption-free measure of discrimination ability, independent of distributional assumptions or fixed thresholds (Hanley & McNeil, 1983; Swets, 1988). Scores range from 0 to 1, with values approaching 1 indicating strong sensitivity, 0.5 representing chance-level performance, and values near 0 reflecting poor discrimination between real and fake images.

**Fig. 2:**
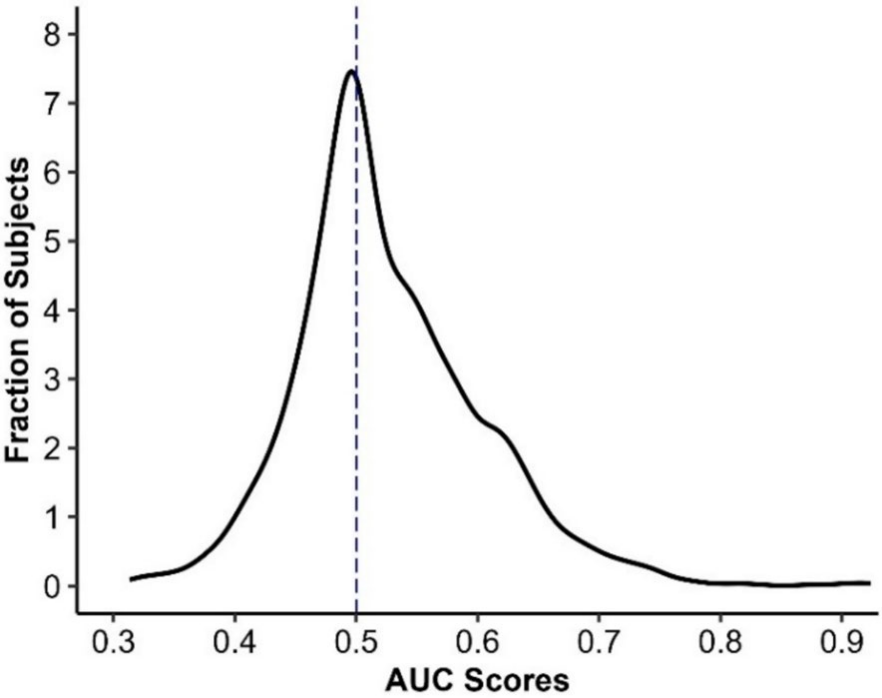
Distribution of AUC scores in humans. The dashed line indicates chance level performance (AUC = 0.50), reflecting no discrimination between deepfake and real face images. AUC = Area Under the Receiver Operating Characteristic Curve

Our findings revealed that the average AUC score was near chance (Fig. 2; *M* = 0.53, *SD* = 0.08, Range = 0.31–0.92), reflecting poor sensitivity in humans to discriminate between deepfake and real images. To examine the extent to which individual differences in psychological variables further predicted discrimination ability we conducted a multiple linear regression model on AUC scores. The statistical model included the main effects of analytic thinking (CRT; continuous), need for cognition (NFC; continuous), positive and negative affect (PANAS, continuous), interoceptive awareness (MAIA-2; continuous), digital literacy (DLS; continuous), and power usage (PUS; continuous), with participant gender, age, and set added as covariates. The overall model was not statistically significant (*R*^*2*^ = 0.01, *F* = 1.22, *p* = .273), with none of the individual difference measures predicting discrimination ability between real and deepfake face images.^3^

#### Machine versus Human Performance

**Fig. 3:**
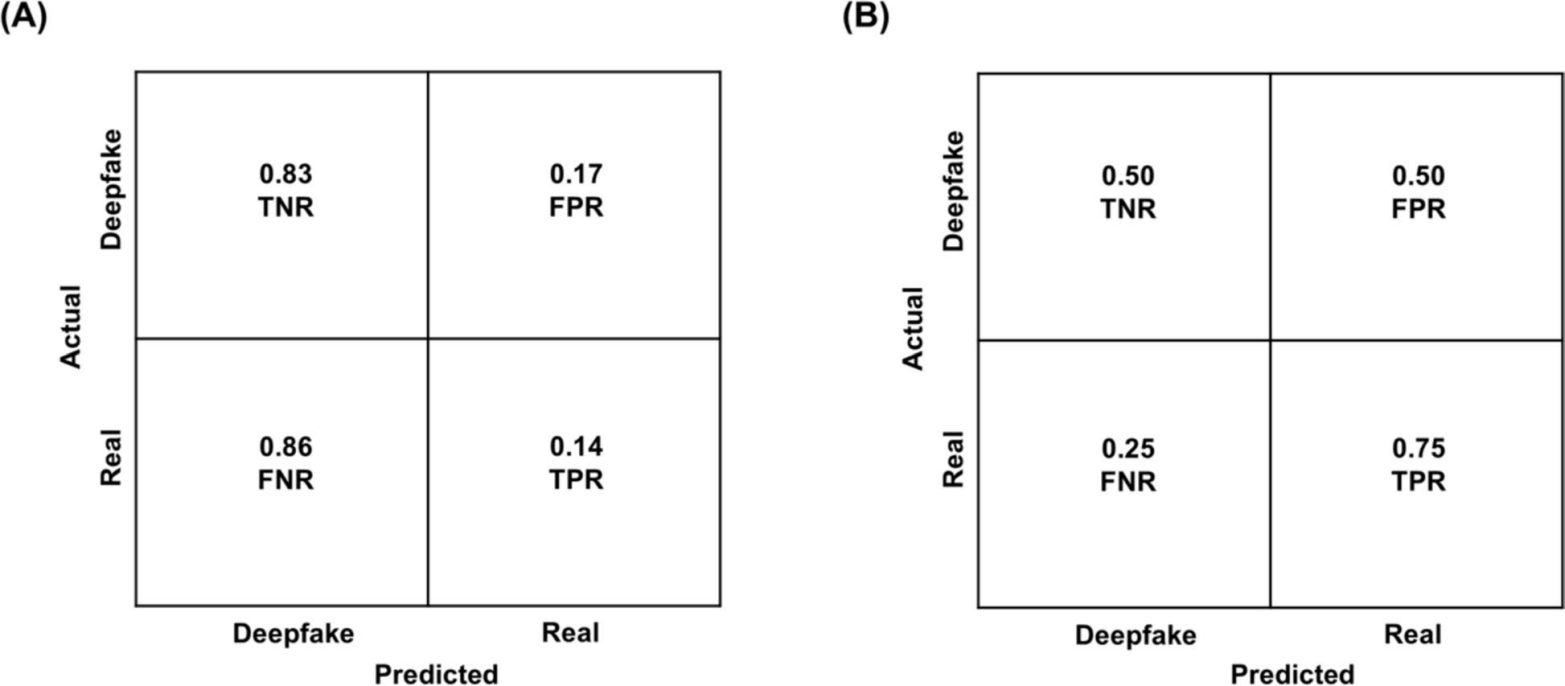
Confusion matrix indicating accuracy for **A** Convolutional Neural Network (CNN) and **B** humans. TNR = True Negative Rate (i.e., correctly classifying deepfake as “deepfake”); FNR = False Negative Rate (misclassifying real as “deepfake”); TPR = True Positive Rate (i.e., correctly classifying real as “real”); FPR = False Positive Rate (i.e., misclassifying deepfake as “real”)

As depicted in the confusion matrix (Fig. 3) for both the CNN algorithm and humans,^4^ we calculated the True Positive Rate (TPR), reflective of the prediction of real when a face image was real; and the True Negative Rate (TNR), reflective of the prediction of fake when a face image was a deepfake. Separate calculation of TPR and TNR for CNN and humans allowed us to determine whether accuracy was comparable for classifications of real and deepfake images or whether it was biased towards one or the other image type (e.g., whether accuracy was high for real images but low for deepfake images). The TPR for the CNN was 97% and the TNR was 97% (Fig. 3A), yielding a 97% overall accuracy (i.e., the mean of TPR and TNR). Thus, the algorithm was equally successful in classifying real and deepfake images, with no detection bias towards one or the other image type. In contrast, the TPR for humans was 67%, whereas the TNR was only 31% (Fig. 3B), with overall accuracy in classifying face images remaining at 49% (i.e., the mean of TPR and TNR). Thus, overall accuracy of humans was lower than overall accuracy of the CNN algorithm. Additionally, the low TNR in humans was driven by a greater tendency to misclassify deepfake images as real (as reflected by a false positive rate (FPR) of 69% in Fig. 3B), suggesting a truth bias in humans (i.e., a tendency to misclassify deepfakes as “real”; AI hyperrealism, Miller et al., 2023).

**Fig. 4:**
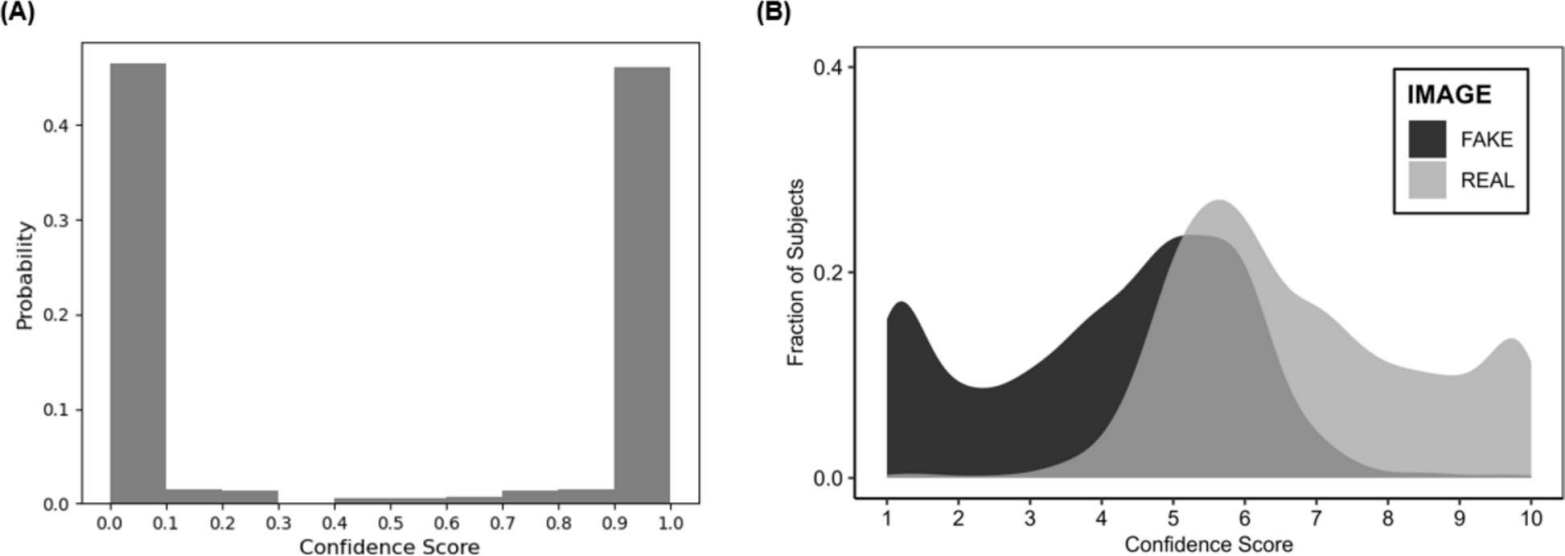
**A** Histogram of probability scores derived from Convolutional Neural Network (CNN) regarding image classification confidence. Scores from 0 to 0.1 reflect higher confidence for classification of real images; scores from 0.9 to 1 reflect higher confidence for classification of deepfake images. The face detection algorithm showed high confidence in both classifying real (i.e., about 45% of scores concentrated around 0 which reflected a higher likelihood that the algorithm classified a face as real) and deepfake (i.e., about 45% of scores concentrated around 1 which reflected a higher likelihood that the algorithm classified a face as fake) images. **B** Distribution of image classification confidence scores in humans. Real images are shown in gray; deepfake images in black. Higher confidence scores reflect higher classification confidence

We also computed decision confidence scores in image classifications for the machine algorithm and the humans. For the CNN algorithm, confidence score was calculated using a probability score derived from the classification prediction. This score represented the confidence level of whether a face classified as real was real on a scale from 0 (*Not confident at all*) to 1 (*Very confident*). Confidence scores assigned by the detection algorithm ranged from 0 and 1, indicating the model’s certainty that a face was fake or real. In particular, scores of (or near) 1 reflected high confidence of the algorithm that a face was fake (i.e., manipulated); a score of (or near) 0 reflected high confidence of the algorithm that a face was real (i.e., genuine). Scores around 0.5 indicated uncertainty, suggesting the algorithm was indecisive about whether a face was fake or real. These intermediate scores typically arise when visual evidence is ambiguous or when the features extracted by the algorithm do not clearly align with those seen in either the one or the other category during training of the algorithm. Ideally, a well-performing model should minimize uncertain scores and produce a bimodal distribution of confidence scores with peaks near 0 and 1. As shown in Fig. 4A, approximately 45% of confidence scores for the CNN fell within 0 and 0.1, indicating high confidence in classifying real images as real. Correspondingly, approximately 45% of the confidence scores were within 0.9 and 1.0, indicating also high confidence in classifying deepfake face images as deepfake. That is, the machine was confident about its prediction. To compute decision confidence in humans on a 1–10 scale (with higher scores reflecting more confidence), we used participants’ original rating responses (1 = *Fake* to 10 = *Real*). In particular, when the actual image was real, confidence scores matched the given rating (e.g., a rating of 10 = confidence of 10; a rating of 1 = confidence of 1). For deepfake images, ratings were reverse-coded so that lower scores indicated higher confidence (e.g., a rating of 1 = confidence of 10; a rating of 5 = confidence of 6). Humans showed higher confidence in classification of real (*M* = 6.77, *SD* = 1.74) than deepfake (*M* = 4.11, *SD* = 1.83) images (*t*(2,202) = 35.95, *p* < .001, Cohen’s *d* = 1.49; Fig. 4B), consistent with their higher accuracy for real than deepfake images.

## Summary and Brief Discussion of Study 1

In Study 1 we found that the CNN approach outperformed the FDA approach in detecting static real and deepfake images, as reflected in greater feature classification accuracy. In comparison, the ability of humans to discriminate between deepfake and real face images was rather poor (near chance level); and individual differences in cognitive and socioemotional processes as well as in the level of internet skills did not explain variability in detection performance for real or deepfake images. Furthermore, our direct comparison between machine and human performance revealed that the CNN algorithm outperformed humans by showing excellent prediction accuracy, with no decision bias and high classification confidence for both deepfake and real face images. Humans’ dramatic underperformance relative to the machine was coupled with a truth bias and low confidence for the classification of deepfake face images.

In this first study, we addressed machine and human performance for deepfake images. Fast-developing AI advances, however, more and more confront us in real life with dynamic deepfakes such as in videos. Importantly, cues available in static vs. dynamic deepfakes differ in that videos often contain audio and visual input simultaneously, integrate behavioral (e.g., facial expressions, gestures) and non-behavioral (e.g., lighting, skin texture) features, and typically are more ecologically valid than static images. Thus, going beyond Study 1, Study 2 examined deepfake detection performance by employing videos *(i)* to investigate sources of misclassification errors in machines, *(ii)* to identify psychological mechanisms underlying detection performance in humans, and *(iii)* to compare humans and machines in their classification decision accuracy and confidence.

## Study 2

### Participants

Study 2 recruited 2,183 undergraduates from the Department of Psychology’s SONA. Of those, 155 who did not continue the study after consenting and 127 who had missing data on one or more of the variables of interest were removed from analysis. The final sample comprised 1,901 participants (Age range: 18–61 years, *M* = 20.26, *SD* = 4.79; 60% female).

### Measures

**Video Rating Task.** Participants viewed 70 short videos of an individual discussing a topic (e.g., book presentations, video games, daily activities). At the end of each video clip, participants were asked to rate the veracity of the face shown in each of the videos on a scale from 100% *(Fake*) to 100% *(Real*), with 50% reflecting just as likely real or fake, which allowed us to capture subtle perceptual differences in the evaluation of the videos. The presentation order of the videos was randomized, and beyond the 10 s video presentation, the task was self-paced.

Videos were obtained from the Deepfake Detection Challenge (DFDC) dataset (Dolhansky et al., 2020), which is a large-scale dataset containing over 100,000 videos, both real and deepfake, covering a variety of scenarios and individuals of diverse gender, age, and racial/ethnic backgrounds. Real videos were created by recording video clips of volunteers. Deepfake videos were generated by applying various manipulation techniques to real videos (e.g., face swapping, altering facial expressions, or audio swapping).

We randomly selected an initial pool of 336 real and 322 deepfake videos. Each video was assessed on multiple criteria to ensure that *(i)* it had a landscape orientation, good sound quality and lighting, and had no text or written information embedded, *(ii)* there was only one person shown in each video, *(iii)* of unique identity (i.e., the same person was not shown in any of the other videos), *(iv)* the person was speaking by looking towards the camera without location change (e.g., walking), and *(v)* videos did not involve audio synthesis or replacement (i.e., audio swapped video) by checking lip syncing. In particular, the final set comprised 35 real and 35 fake videos, all trimmed to 10 s to ensure equal duration. To assure that detection performance was not confounded by audio in the videos, the same set of videos were muted to create non-audio video versions. For counterbalancing, approximately half of the participants (N = 897) viewed the videos with audio and approximately the other half (N = 1,004) viewed the muted versions, with videos presented in random order in each of these two stimuli lists. All videos are achieved under the OSF repository (https://osf.io/qhm3y/?view_only=bdc41a53bf7a4367bde6951372d9c932).

### Procedure

Study procedures were approved by the University of Florida Institutional Review Board (IRB# 202,102,022). Identical to Study 1, participants consented electronically and completed the study remotely through Qualtrics. Participants first completed the Video Rating Task, followed by the CRT, NFC, MAIA-2, PANAS, DLS, PUS, and a brief demographic questionnaire, in this order. The study took approximately 100 min and participants were reimbursed with SONA credits upon completion.

### Analyses and Results

All de-identified datasets and analysis scripts used in Study 2 are available on the OSF repository (https://osf.io/qhm3y/?view_only=bdc41a53bf7a4367bde6951372d9c932).

#### Machine Performance

To measure how well machines detect video deepfakes, we tested two different ML algorithms: The first was FaceForensics (using a pre-trained Xception network; Rössler et al., 2019); the second involved Recurrent Neural Network (RNN) (using the pre-trained network; Güera & Delp, 2018).^5^ We specifically selected these two video detection algorithms because they are known to be efficient in identifying inconsistencies and manipulations of latent features from continuous frames. Additionally, employing these ML algorithms ensured independence of test performance from the training process as they were not trained on the DFDC dataset from which our videos were drawn. As in Study 1, predicted labels generated by the ML algorithms were either 0 = Deepfake or 1 = Real face and reflected the classification for each face type within the videos. FaceForensics yielded 49% accuracy in distinguishing real and deepfake videos, whereas RNN resulted in 39% accuracy.

**Fig. 5:**
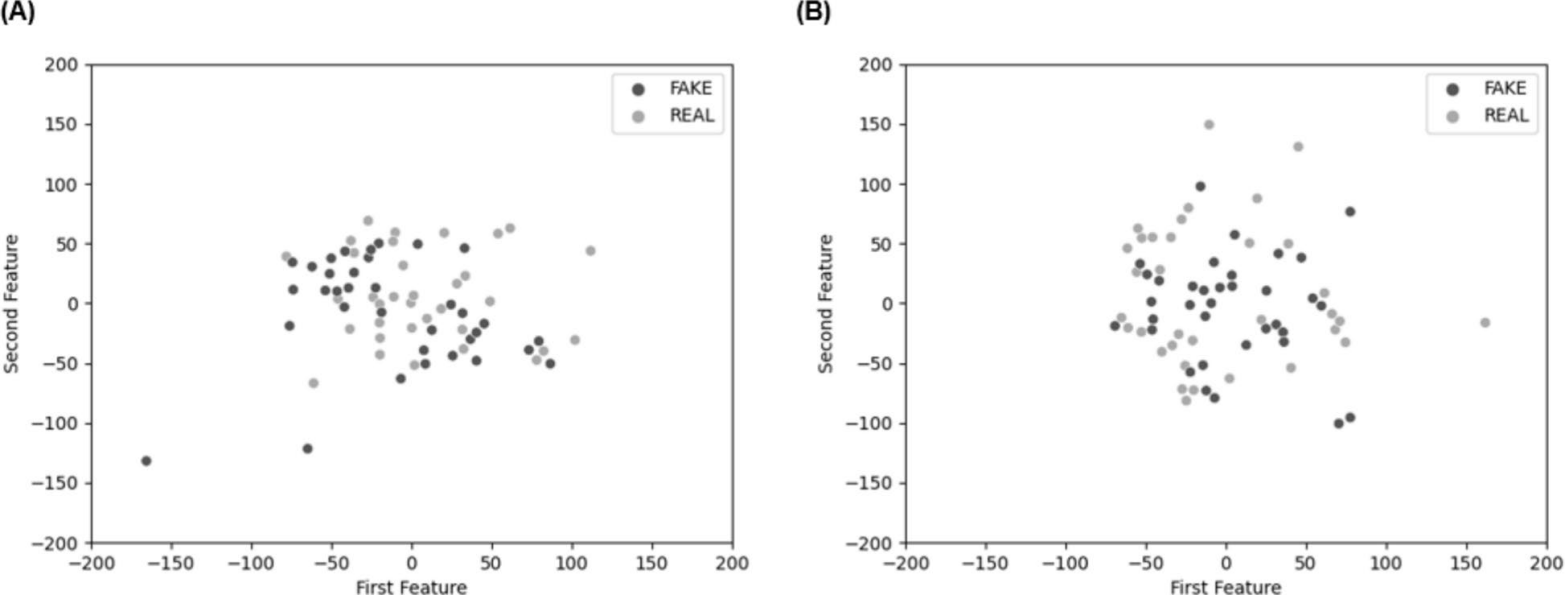
2-D visualization of latent features from **A** FaceForensics and **B** Recurrent Neural Network (RNN). Real videos are shown in gray, deepfake videos in black

To identify the source of misclassification, we applied the same feature visualization technique as in Study 1. Features were intertwined for both FaceForensics (Fig. 5A) and RNN (Fig. 5B), making it difficult to establish a clear decision boundary and resulting in poor classification of features for both ML algorithms. Of note, RNN misclassified features even more than FaceForensics, possibly because the RNN algorithm uses the entire frame to extract features, whereas the FaceForensics model uses a frontal face frame only.

#### Human Performance

**Fig. 6:**
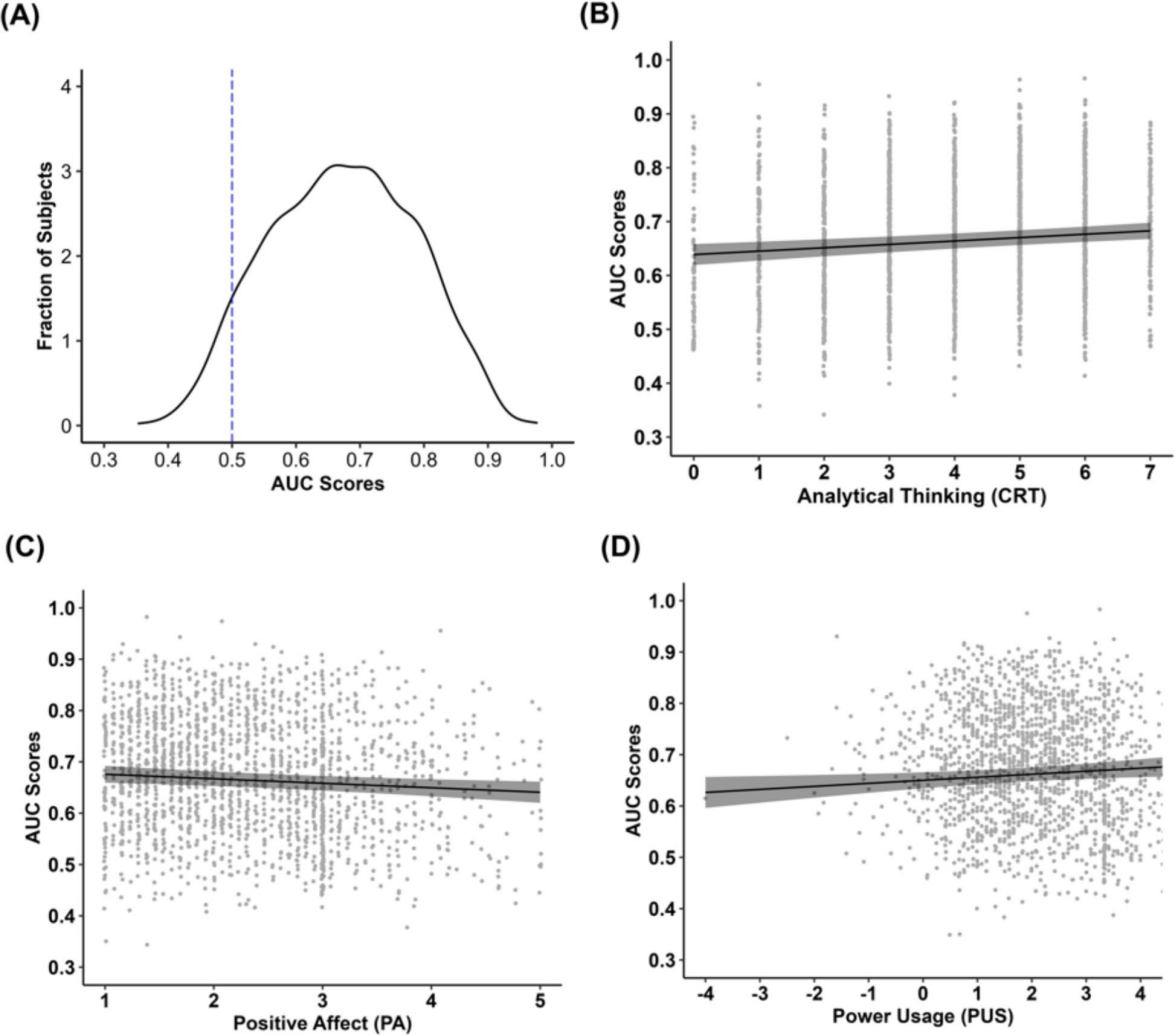
**A** Distribution of AUC scores in humans. The dashed line indicates chance level performance (AUC = 0.50), reflecting no discrimination between deepfake and real videos. AUC = Area Under the Receiver Operating Characteristic Curve. Greater discrimination between deepfake and real videos was associated with **B** higher analytical thinking, indexed by Cognitive Reflection Test (CRT) scores, **C** lower positive affect, indexed by Positive and Negative Affect Scale (PANAS) scores, and **D** greater power usage, indexed by Power User Scale (PUS) scores. Each dot represents a participant. Shaded areas around the regression lines reflect the 95% confidence interval

As in Study 1, we first assessed the video rating data for normality and homogeneity of variances. Shapiro–Wilk tests indicated significant deviations from normality for both real and fake videos (*W*s > 0.92, *p*s < 0.01). Additionally, an F-test revealed unequal variances between real and fake faces (*F*(50,469, 50,411) = 1.34, *p* < 0.001). Given these violations, we again employed non-parametric AUC scores (Hanley & McNeil, 1983) derived from the participants’ continuous ratings as an index of sensitivity in discriminating between real and deepfake videos. The ability to discriminate between deepfake and real videos was fairly good in humans (Fig. 6A; *M* = 0.67, *SD* = 0.11, Range = 0.35–0.98). We also again conducted a multiple linear regression on AUCs for formal analysis as in Study 1. This statistical model again included the main effects of analytic thinking (CRT; continuous), need for cognition (NFC; continuous), positive and negative affect (PANAS, continuous), interoceptive awareness (MAIA-2; continuous), digital literacy (DLS; continuous), and power usage (PUS; continuous), with participant gender, age, and video modality added as covariates. The overall regression model was statistically significant and accounted for approximately 10% of the variance in AUC scores (*R*^*2*^ = 0.10, *F* = 13.31, *p* < .001). Specifically, greater ability to discriminate between deepfake and real videos was associated with higher analytical thinking (reflected by a significant main effect of CRT: *β* = 0.006, *F* = 4.32, *p* < .001, Cohen’s *f*^2^ = 0.01; Fig. 6B), lower positive affect (reflected by a significant main effect for PA: *β* = − 0.009, *F* = 2.85, *p* = 0.011, Cohen’s *f*^2^ = 0.005; Fig. 6C), and greater power usage (reflected by a significant main effect for PUS: *β* = 0.006, *F* = 2.43, *p* = 0.032, Cohen’s *f*^2^ = 0.003; Fig. 6D). None of the other individual difference variables significantly predicted discrimination ability (all *F*s < 2.12, *p*s > 0.07).

#### Machine versus Human Performance

**Fig. 7:**
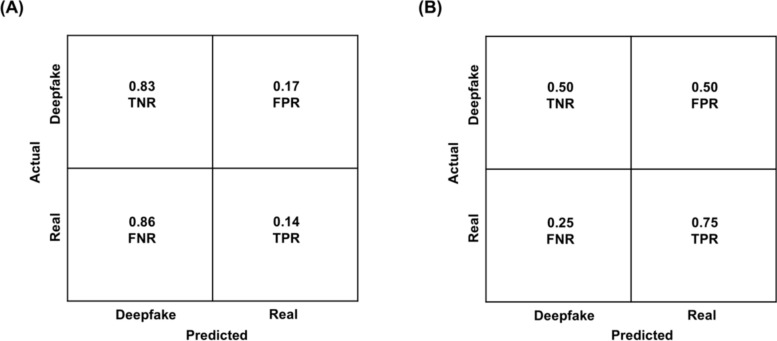
Confusion matrix indicating accuracy for **A** FaceForensics and **B** humans. TNR = True Negative Rate (i.e., correctly classifying deepfake as “deepfake”); FNR = False Negative Rate (misclassifying real as “deepfake”); TPR = True Positive Rate (i.e., correctly classifying real as “real”); FPR = False Positive Rate (i.e., misclassifying deepfake as “real”)

As depicted in the confusion matrix (Fig. 7) for both the CNN algorithm and humans^6^ and as in Study 1, we calculated the TPR (i.e., the prediction of real when a video was real) and TNR (i.e., the prediction of fake when a video was a deepfake). The TNR for FaceForensics was 83%, whereas the TPR was only 14% (Fig. 7A), yielding 49% overall accuracy (i.e., the mean of TPR and TNR scores) in classifying videos. The low TPR for FaceForensics was driven by a greater tendency to misclassify real videos as deepfake (i.e., lie bias), as reflected by an FNR of 86% (Fig. 7A). The TNR for humans was 50%, whereas the TPR was 75% (Fig. 7B), with 63% overall accuracy (i.e., the mean of TPR and TNR) in classifying videos. Thus, different from the results for static images, humans outperformed the FaceForensics algorithm in classifying videos.

**Fig. 8:**
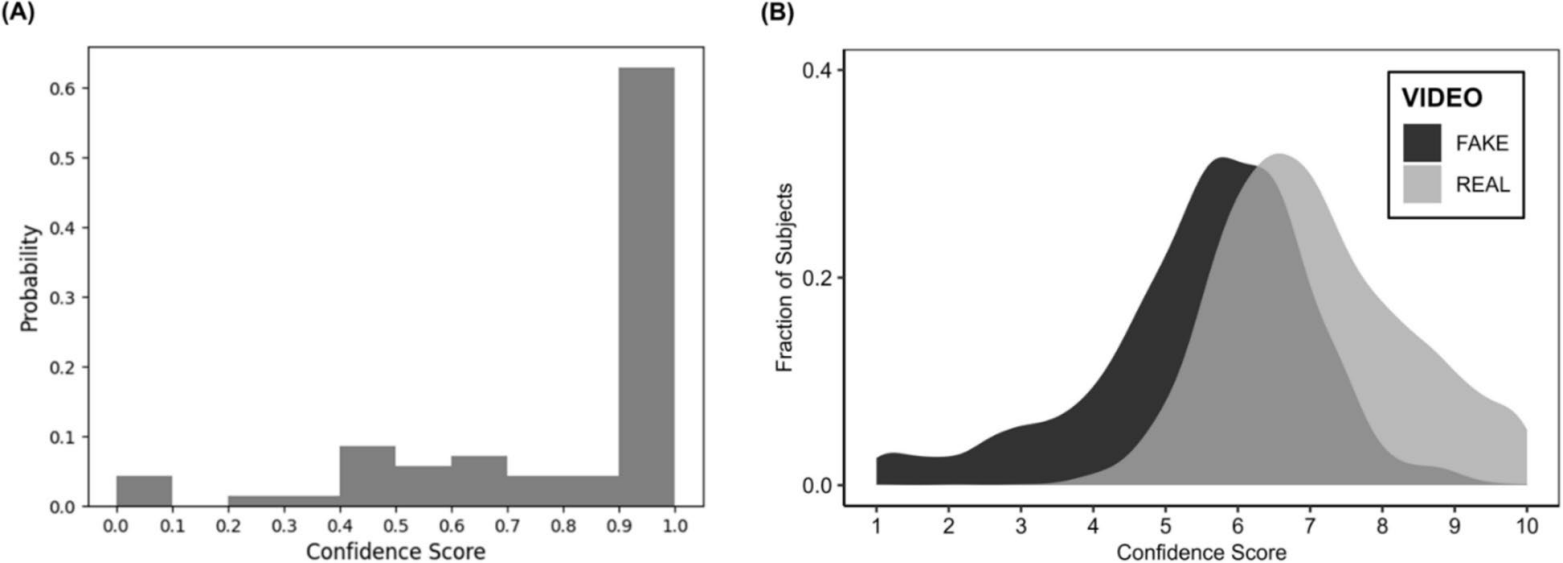
**A** Histogram of probability scores from FaceForensics for video classification confidence. Scores from 0 to 0.1 reflect higher confidence for classification of real videos; scores from 0.9 to 1 reflect higher confidence for classification of deepfake videos. A greater portion of the confidence scores (63%) gathered around 1, which reflected higher confidence of the algorithm in its classification of a video as fake. The remaining 37% of confidence scores were equally distributed across other bins reflecting indecisiveness of the algorithm about classifying a video as real. **B** Distribution of video classification confidence scores in humans. Real videos are shown in gray, deepfake videos in black. Higher confidence scores reflect higher confidence

Parallel to Study 1, we again computed decision confidence scores in video classifications for both the machine and humans. For FaceForensics (Fig. 8A), up to 63% of the confidence scores fell within the range of 0.9 to 1.0, while the remaining 37% were equally distributed across other bins. This pattern confirmed the algorithm’s lie bias and suggests that it was uncertain about its decisions while classifying a video as "real". For humans (Fig. 8B), confidence in the classification of real videos (*M* = 7.05, *SD* = 1.29) was higher than confidence in the classification of deepfake videos (*M* = 5.51, *SD* = 1.52; *t*(1,900) = 27.85, *p* < .001, Cohen’s *d* = 1.10), consistent with the humans’ higher accuracy for real than deepfake videos.

## Summary and Brief Discussion of Study 2

In Study 2 we found that while the FaceForensics algorithm performed slightly better than the RRN algorithm at detecting real and deepfake videos, accuracy for FaceForensics was rather low (near chance level). We observed that classification accuracy of features in videos was intertwined and poor for both ML algorithms. In contrast, discrimination ability between deepfake and real videos in humans was rather good. Further, higher analytical thinking, less positive affect, and greater internet skills were associated with better discernment ability. Directly comparing machine and human performance, furthermore, showed that the overall classification accuracy of FaceForensics was lower than the human performance, with this underperformance by the machine characterized by a lie bias and low classification confidence for real videos. A decision bias was less evident in humans, with decision confidence patterns in alignment with detection accuracy for real and deepfake videos.

## General Discussion

With rapidly increasing sophistication of AI, deepfakes represent a serious challenge in today’s society. They are being used to deceive and disseminate disinformation, undermining trust in media and institutions. While research on deepfake detection performance in both machines and humans is growing, the processes underlying deepfake detection ability are not well understood; and direct comparisons of machine vs. human performance are still rare. Here we identified sources of misclassification errors in machines, psychological mechanisms of discrimination ability in humans, and directly contrasted machine and human performance regarding classification accuracy and confidence for real and deepfake images (Study 1) and videos (Study 2). Across two studies, our data yielded three key findings: First, ML algorithms were overall more accurate and better at classifying features in real and deepfake images than videos. Second, humans outperformed the ML algorithm in deepfake video detection, but they experienced challenges in deepfake image detection, where they displayed a truth bias (i.e., AI Hyperrealism; Miller et al., 2023) and low confidence. In turn, the ML algorithm’s quite weak performance with videos was marked by a lie bias and low classification confidence. Third, we found that higher analytical thinking, lower positive affect, and more internet skills improved discernment of deepfake from real videos in humans. Collectively, these findings suggest that ML excels at detecting deepfake images (static input) but humans have an advantage in video detection (dynamic input). This differential pattern of findings highlights the need for collaboration between humans and AI to optimize the detection of deepfakes. Theoretical and practical implications of our novel findings are discussed next.

### Machines Excel in Image Deepfake Detection but Experience Challenges with Videos

CNN and FDA ML algorithms achieved high detection accuracies of 97% and 79%, respectively, for image deepfakes (Study 1). Follow-up feature space analysis further demonstrated that CNN was more effective at discernment than FDA because features learned by this algorithm clustered more tightly for both deepfake and real images. The scattering of features in FDA compared to the more tightly clustered features learned by CNN may have stemmed from their different approaches to feature selection. That is, CNN is trained to identify distinctive features from both deepfake and real images. FDA, in contrast, produces more dispersed features from real images because it relies on the Fourier transform to detect unique patterns specific to deepfake (but not real) images. This, in turn, leads to less accurate real image classification.

In contrast, video deepfake detection by FaceForensics and RNN algorithms (Study 2) achieved low accuracies of 49% and 39%, respectively. Follow-up feature space analysis for these algorithms revealed that both methods struggled with the identification of distinctive features that effectively differentiated between deepfake and real videos. Visualization of this performance pattern revealed that features from real and deepfake videos were entangled and indistinguishable, leading to classification error. Our finding that FaceForensics outperformed the RNN in deepfake detection may be attributed to differences in how these algorithms operate. Specifically, the deepfake videos in the DFDC dataset were generated using face-swapping techniques, a method that FaceForensics is particularly well-suited to detect. In contrast, the RNN algorithm processes entire video frames for feature extraction (Güera & Delp, 2018), This broader frame-level analysis may have led to more misclassifications, as the manipulations in the DFDC videos are confined to the facial region, leaving the background untouched. In particular, in the videos used here, only the facial region was modified, while the background remained unaltered. This selective manipulation presents a significant challenge for detection algorithms, particularly when the face is small within the overall video frame—meaning the algorithm processes a larger context where the manipulation is not present. As a result, the altered facial features were more subtle and thus may have been harder to detect, diminishing the algorithm’s ability to identify the tampering. Consequently, the algorithm may have misclassified the deepfakes as real, influenced by the prominence of unaltered background content and the only small, relatively restricted area of the modified face.

### Machines Outperform in Image Detection but Humans Lead in Video Deepfake Detection

Our comparison of machine and human performance for classifying face images (Study 1) found that the CNN algorithm outperformed human detection ability, showing excellent accuracy without decision bias and maintaining high confidence. In contrast, humans performed significantly worse, with overall accuracy at chance level. Humans also showed a truth bias in their decision criteria, reflected in a greater tendency to misclassify deepfake images as real, and this bias was accompanied by low deepfake image classification confidence. These findings suggest that sophisticated modern ML models can generate deepfake face images that are indistinguishable from real face images in the eye of human perceivers.

Regarding classification of deepfake videos (Study 2), however, we found that humans outperformed the FaceForensics algorithm in overall accuracy, and the machine showed a lie bias and low classification confidence. In contrast, humans’ greater accuracy and reduced decision bias when classifying deepfake videos than images, and also relative to the performance of the machine, suggest that rich perceptual cues in dynamic stimuli (e.g., motion and temporal consistency) facilitate deepfake detection in humans; whereas ML algorithms are less able to benefit from such cues.

This differential pattern of findings for images vs. videos point out that humans and machines employ rather different mechanisms in deepfake detection, highlighting the potential for human-AI collaboration to optimize performance (e.g., by supporting human decision making with machine predictions and by feeding human-perceived cues/features to improve an algorithm’s prediction; Groh et al., 2022; Miller et al., 2023). Along these lines, future research could use two-alternative forced choice designs, where a deepfake face image is presented alongside its corresponding real face while recording eye movements of human perceivers. This approach would allow researchers to identify erroneous visual viewing patterns and capture attention to non-diagnostic cues in humans, and this could then be followed up with AI-facilitated eye tracking training, in which diagnostic features deemed as critical by ML for deepfake detection are targeted for guiding human attention and processing.

### Higher Analytical Thinking, Lower Positive Affect, and Greater Internet Skills Predict Better Video Deepfake Detection

Results from Study 2 suggest that higher analytical thinking, less positive affect, and greater internet skills were associated with better discernment of deepfake from real videos. Analytical thinking has emerged as a reliable predictor of fake news detection (Bago et al., 2020; Pehlivanoglu et al., 2021, 2022; Pennycook & Rand, 2019). Extending this work to deepfakes here for the first time, our findings suggest that elaborative, relative to shallow, processing may foster attention to spot digital manipulations (e.g., face swapping) in video deepfakes. We also found that less positive affect was related to greater discernment between deepfake and real videos. This finding is in line with evidence that less positive affect enhances deliberative decision making (Schwarz & Clore, 2003) and deception detection (Matovic et al., 2014; but see Ebner et al., 2020). Finally, higher power usage was related to better ability to distinguish between deepfake and real videos. There is previous evidence showing that time spent on social media was linked to less susceptibility to fake news (Halpern et al., 2019) and deepfake videos (Nas & de Kleijn, 2024). Our measure on power usage went beyond previous operationalizations, which solely assessed time spent on social media, by considering and demonstrating the role of prior experience, expertise, and self-efficacy pertaining to technology use on video deepfake detection.

### Limitations and Future Directions

All algorithms used in this work were originally pre-trained by their developers. To maintain evaluation integrity and fairness, we deliberately excluded some algorithms (e.g., GenConViT; Deressa et al., 2023) that were trained on the same datasets (i.e., DFDC) that included our test samples. This measure was taken to avoid an overlap between training and testing data, preventing data leakage, and ensuring independence of test performance from the training process. However, we recognize that this decision may have limited the potential of the selected ML models to reach their best possible performance, as many state-of-the-art algorithms benefit significantly from extensive stimulus-specific training. Thus, our findings may not reflect how well these models could have performed under ideal, task-tailored conditions. Rather, one of the primary goals of this study was to test how well performance of existing ML algorithms generalize to unseen content (instead of engineering optimal solutions) along with comparing ML performance to human detection ability.

Our finding of low machine classification accuracy for videos may appear inconsistent with previous research on fake video detection (Jung et al., 2020; Li et al., 2018; Matern et al., 2019; Yang et al., 2018), but it is important to consider key methodological differences across studies. These previous studies employed biologically based detection algorithms that depended on high-resolution, high-quality video data to accurately extract subtle cues such as eye movements, micro-expressions, and physiological signals. While the datasets used here were not optimized for capturing this level of detail, future research should investigate whether these advanced detection models can maintain their performance when applied to lower-resolution, ecologically valid stimuli that reflect real-world conditions.

Also, our rating scales slightly differed across studies to accommodate for task-specific features (i.e., static faces in Study 1, dynamic videos in Study 2). These differential formats, however, may have encouraged judgments based on detection confidence (“*How certain am I that this is real or fake?*”) vs. perceived stimulus quality (“*How much artificiality does this stimulus contain?*”), and thus have been reflective of distinct cognitive processes. This methodological variability across our studies somewhat limits direct cross-study comparability. Future research can address this limitation by employing harmonized response formats and, where possible, two-step rating procedures (e.g., binary real/fake judgments followed by separate confidence ratings; Macmillan & Creelman, 1991) to capture and isolate the respective psychological constructs.

Further, in the current work, each participant rated a large number of unique real and fake stimuli only once, preventing the computation of intra-rater reliability, which constitutes an important psychometric indicator of the stability of judgments across repeated exposures. Although this design choice was necessary to avoid fatigue given the large number of unique stimuli, it limits our ability to determine whether the lack of associations between individual differences and detection performance in Study 1 may have, at least partly, resulted from low within-person consistency. Future research should incorporate repeated item designs to directly quantify intra-rater reliability and assess whether individual differences emerge when measurement precision is increased.

Moreover, the StyleGAN static face images used in Study 1 were generated through random sampling from Gaussian noise. Therefore, demographic attributes such as age and race could not be experimentally controlled, and the resulting face image pool reflected the demographic biases of the FFHQ training dataset. Indeed, a post-hoc demographic classification analysis using the DeepFace framework (Serengil & Ozpinar, 2021) revealed an imbalanced distribution of demographic features of our faces in Study 1 (mean estimated age = 31.7 years; approximately 57% White, 17% Asian, 6% Black, and 20% Other), which may have influenced deepfake detection performance. This variability is relevant in light of emerging evidence that human detection performance may differ for real and fake faces from specific demographics (e.g., between white real and fake faces; Miller et al., 2023). Moving forward the use of generative approaches that enable explicit demographic control of facial stimuli (e.g., conditional GAN architectures; Choi et al., 2020; Mirza & Osindero, 2014; Xu et al., 2018) or use of curated, demographically balanced face datasets to minimize representational bias and allow systematic examination of demographic effects on deepfake detection will be beneficial.

Finally, the literature suggests that older adults may be more vulnerable to certain types of deception, including digital misinformation (Pehlivanoglu et al., 2022), phishing (Ebner et al., 2020; Pehlivanoglu et al., 2024), and lie detection (Ruffman et al., 2012). These findings point to the need for extending the current work to aging populations, especially given evidence that older adults face unique challenges in detecting deceptive content online (Ebner et al., 2023). Investigating deepfake susceptibility in older age demographics will not only clarify the role of age in media judgment but will also inform the design of age-tailored interventions to enhance digital literacy and resilience against visual misinformation.

## Conclusions

Across two studies, employing deepfake images and videos, and directly comparing human and machine performance, we found that ML algorithms have superior accuracy and better feature classification for real and deepfake static images than dynamic videos. The machines’ underperformance for videos was accompanied by a lie bias and low classification confidence for deepfake videos. We also found that humans outperformed ML algorithms in deepfake video detection; while they performed only at chance level in detecting deepfake images, for which they displayed a truth bias and low decision confidence. We also provide first evidence that higher analytical thinking, less positive affect, and greater internet skills are conducive to better discernment between real and deepfake videos among humans. These findings combined importantly advance understanding of the mechanisms involved in deepfake detection, delineating conditions under which human–machine collaboration may be particularly fruitful, to jointly combat this significant threat.

### Significance statement

Deepfake technology represents a growing threat to information authenticity, challenging both humans and machine learning (ML) systems. This study provides a comprehensive comparison of human and ML performance in detecting static and dynamic visual deepfakes, highlighting their respective strengths and weaknesses. While ML algorithms excel at detecting static deepfake face images with high accuracy, humans struggle, exhibiting a truth bias and low decision confidence. Conversely, humans outperform ML systems in detecting dynamic video deepfakes, leveraging analytical thinking, mood, and internet skills. These findings underscore the need for human-AI collaboration to enhance deepfake detection capabilities. By identifying cognitive and technical factors that improve human detection performance and pinpointing areas where ML or humans struggles, this research offers actionable insights for developing robust, interdisciplinary solutions to counter the growing threat of deepfakes.

## Supplementary Information

Additional file1.

## Data Availability

The full set of de-identified datasets, analysis code, and materials from Study 1 and 2 are available on OSF at https://osf.io/qhm3y/?view_only=bdc41a53bf7a4367bde6951372d9c932.

## Footnotes

1 The number of participants assigned to each set was: Set 1 = 737; Set 2 = 736; Set 3 = 730.

2 The CNN model (Wang et al., 2020) was pre-trained to distinguish ProGAN-generated (Karras et al., 2017) objects from real scenes in the Large-scale Scene Understanding (LSUN) dataset (Yu et al., 2015). This model transfers well to the static faces used in Study 1 as it can detect low-level frequency artifacts independent of image semantics. The FDA-based classifier (Durall et al., 2019) was trained on the Faces-HQ dataset (combining CelebA-HQ, FFHQ, and their GAN counterparts; Durall et al., 2019), which aligns closely with our StyleGAN2 (Karras et al., 2020) deepfake static images derived from FFHQ (Karras et al., 2019).

3 To account for multiple comparisons involving individual difference variables, a false discovery rate (FDR) correction was applied to ensure rigorous control of Type I error in Study 1 and 2. Correlations between the key study variables in Study 1 and 2 are presented in the Supplemental Results.

4 To create a confusion matrix for human data, we dichotomized responses on the face image rating scale ranging from 1 (*Fake*) to 10 (*Real*). For deepfake images, ratings from 1 to 5 reflected TNR; ratings from 6 to 10 reflected FPR. For real face images, ratings from 1 to 5 reflected FNR; ratings from 6 to 10 reflected TPR.

5 The XceptionNet (Rössler et al., 2019) was pre-trained on a corpus of 1,000 original video sequences manipulated via four automated methods (Deepfakes, Deepfake, 2018; Face2Face, Thies et al., 2016; FaceSwap, Kowalski, 2018; NeuralTextures, Thies et al., 2019). The RNN model (Güera and Delp, 2018) was developed using a custom dataset of 300 self-generated deepfakes with FaceSwap (Deepfake, 2018) and 300 real videos from the Hollywood Human Actions (HOHA) dataset (Laptev et al., 2008). While these pre-training datasets are less extensive than the DFDC dataset used in our Study 2, both models were trained specifically on manipulated facial video content, providing a valid baseline for detecting spatial artifacts (e.g., blending boundaries) and temporal inconsistencies (e.g., inter-frame flicker).

6 To create a confusion matrix for human data, we dichotomized responses to the video rating scale ranging from 100% (Fake) to 100% (Real). For deepfake videos, ratings from 100% (Fake) to 60% (Fake) reflected ‘TNR’; ratings from 100% (Real) to 60% (Real) reflected ‘FPR’. For real videos, ratings from 100% (Real) to 60% (Real) reflected ‘TPR’; ratings from 100% (Fake) to 60% (Fake) reflected ‘FNR’. Responses of 50% were omitted from the analysis (8% of the trials).
