# Supplementary material for: Is this real? Susceptibility to deepfakes in machines and humans
Source: Cogn Res Princ Implic. 2026 Jan 7;11:3. doi: 10.1186/s41235-025-00700-y (PMC12779810; doi:10.1186/s41235-025-00700-y)
Supplement: Supplementary file 1 — Additional file1. [file 41235_2025_700_MOESM1_ESM.docx]

**Supplemental Results**

Table S1

*Correlations between the key study variables in Study 1*

| **Variable** | **CRT** | **NFC** | **PA** | **NA** | **MAIA** | **DL** | **PUS** |
| --- | --- | --- | --- | --- | --- | --- | --- |
| **CRT** | 1 |  |  |  |  |  |  |
| **NFC** | 0.18*** | 1 |  |  |  |  |  |
| **PA** | -0.14*** | 0.13*** | 1 |  |  |  |  |
| **NA** | -0.19*** | -0.16*** | 0.25*** | 1 |  |  |  |
| **MAIA** | -0.04 | 0.19*** | 0.30*** | -0.09*** | 1 |  |  |
| **DL** | 0.12*** | 0.16*** | 0 | -0.10*** | 0.17*** | 1 |  |
| **PUS** | 0.05* | 0.04* | -0.01 | -0.13*** | 0.17*** | 0.34*** | 1 |

***Note:*** CRT: Cognitive Reflection Test; NFC: Need for Cognition; PA: Positive Affect; NA: Negative Affect; MAIA: Multidimensional Assessment of Interoceptive Awareness; DL: Digital Literacy; PUS: Power User Scale. * *p* < .05; ** *p* < .01; *** *p* < .001

Table S2

*Correlations between the key study variables in Study 2*

| **Variable** | **CRT** | **NFC** | **PA** | **NA** | **MAIA** | **DL** | **PUS** |
| --- | --- | --- | --- | --- | --- | --- | --- |
| **CRT** | 1 |  |  |  |  |  |  |
| **NFC** | -0.06* | 1 |  |  |  |  |  |
| **PA** | -0.18*** | 0.03 | 1 |  |  |  |  |
| **NA** | -0.23*** | 0.08*** | 0.30*** | 1 |  |  |  |
| **MAIA** | -0.02 | 0.01 | 0.29*** | -0.08*** | 1 |  |  |
| **DL** | 0.14*** | -0.04 | 0.02 | -0.07** | 0.19*** | 1 |  |
| **PUS** | -0.18*** | 0.06** | 0.22*** | 0.27*** | 0 | 0.18*** | 1 |

***Note:*** CRT: Cognitive Reflection Test; NFC: Need for Cognition; PA: Positive Affect; NA: Negative Affect; MAIA: Multidimensional Assessment of Interoceptive Awareness; DL: Digital Literacy; PUS: Power User Scale. * *p* < .05; ** *p* < .01; *** *p* < .001
